# Microfluidic Approaches to Pickering Emulsions and Foams: Strategies, Challenges, and Promising Applications

**DOI:** 10.1002/smll.202507603

**Published:** 2025-10-29

**Authors:** Xuefeng Shen, Fathinah Islami Hasyyati, Karin Schroën, Jasper van der Gucht, Siddharth Deshpande

**Affiliations:** ^1^ Laboratory of Physical Chemistry and Soft Matter Wageningen University and Research Stippeneng 4 Wageningen 6708 WE The Netherlands; ^2^ Laboratory of Food Process Engineering Wageningen University and Research Bornse Weilanden 9 Wageningen 6708 WG The Netherlands

**Keywords:** colloidal particles, interface, microfluidics, pickering emulsions, pickering foams

## Abstract

Particle‐stabilized emulsions and foams, commonly referred to as Pickering emulsions and foams, offer superior stability and greater functional versatility compared to their conventional polymer‐ or surfactant‐stabilized counterparts, bestowing them with unique features. However, the understanding of particle adsorption dynamics, particle‐droplet (or particle‐bubble), and interdroplet (or interbubble) interactions during large‐scale emulsification remains limited, hindering full exploitation of their potential. In recent years, on‐chip microfluidic techniques have provided an effective experimental platform to precisely design and produce Pickering emulsions, and perform systematic analyses with regards to their formation, stability, and dynamics. This review examines recent microfluidic advances in the production and analysis of Pickering emulsions and foams. The discussion focuses on their underlying working principles and classification of the current methods based on the mechanisms by which particles are loaded onto interfaces. The review concludes with a critical evaluation of the advantages, limitations, and emerging applications of microfluidic strategies in this field. Looking ahead, this review highlights how the integration of microfluidics with advanced materials science and analytical tools is expected to open new opportunities for designing functional interfacial systems, enabling process scale‐up, and translating Pickering systems into practical applications.

## Graphical abstract

Microfluidic techniques offer a precise platform for designing and analyzing Pickering emulsions and foams. This review examines key advancements in their production, characterization, and potential applications, while also addressing key challenges and future prospects in utilizing microfluidics for these complex colloidal systems. Image created using BioRender.com.

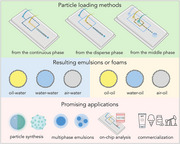

## 1 Introduction

Emulsions consist of liquid droplets dispersed within an immiscible continuous liquid phase. Foams share a similar structure, but the dispersed phase is made up of gas bubbles encased by a thin, continuous liquid film. Emulsions and foams widely occur in various industries, including food, cosmetics, pharmaceuticals, oil recovery, and wastewater treatment.^[^ ^1^ ^]^ These systems are typically thermodynamically unstable due to the high interfacial energy between the dispersed phase (droplets in case of emulsions and bubbles in case of foams) and the continuous phase (liquids). This leads to a tendency to reduce the total free energy by increasing droplet or bubble size, consequently decreasing the total interfacial area, and leading to physical destabilization through mechanisms such as coalescence and Ostwald ripening.^[^ ^2^ ^]^ Maintaining the stability of foams and emulsions is crucial as it influences the properties of the prepared materials in time. Commonly, surfactants and amphiphilic polymers are employed to stabilize these systems by lowering the interfacial tension, creating layers around droplets or bubbles, and preventing coalescence.^[^ ^3^ ^]^ However, these stabilizers often require higher concentrations and are sensitive to changes in pH, temperature, and ionic strength.^[^ ^4^ ^]^

In addition to traditional surfactants and amphiphilic polymers, emulsions and foams can also be stabilized by particles. The stabilizing effect of solid particles on emulsions and foams was first realized in the early 20th century and such emulsions were named after the British chemist, S. U. Pickering.^[^ ^5^ ^]^ It is worth noting that the adsorption of particles on an interface is essentially an irreversible process. The energy required to detach an adsorbed particle from the interface can be calculated as Δ*G* = −γπ*r* ^2^(1 − |cos θ|)^2^, where *r* is the particle radius, γ the interfacial tension between the two phases and θ the three phase contact angle of the particles at the interface. Correspondingly, for particles larger than a few nanometres, and θ not too close to 0° or 180°, the values of Δ*G* greatly exceed the thermal energy *k*_*B*_ *T*, virtually making it an irreversible process.^[^ ^6^, ^7^ ^]^ This is in stark contrast to surfactants, which can detach from the interface owing to thermal fluctuations and the chemical nature of the surfactant‐fluid interaction. This irreversible adsorption of particles creates a steric barrier, preventing the coalescence and Ostwald ripening of droplets and bubbles.^[^ ^2^ ^]^ Consequently, particle‐stabilized emulsions and foams—commonly known as Pickering emulsions and foams—are increasingly recognized for their ability to provide long‐term stability with minimal or no use of surfactants.^[^ ^8^, ^9^, ^10^ ^]^

Conventional emulsification techniques such as rotor‐stator homogenization, high‐pressure homogenization, and sonication are commonly used to produce Pickering emulsions and foams. However, these methods often yield a broad droplet size distribution.^[^ ^11^ ^]^ Moreover, it is not possible to separate the breakup, transport, and coalescence of droplets, as well as the particle loading process in such bulk emulsification methods, complicating the isolation of individual effects such as particle shape, pH, salt concentration, and the presence of surfactants.^[^ ^12^, ^13^ ^]^ Furthermore, the emulsification process may be detrimental to the particles, breaking them up as well.^[^ ^14^ ^]^

To deconvolute these effects, microfluidic techniques hold unique advantages. Microfluidics encompasses a collection of tools designed for manipulating fluids and materials typically at the scale of a few to hundreds of microns.^[^ ^15^ ^]^ Microfluidic emulsification offers a precise control over production parameters (production rate, droplet size, etc.) and further interactions, significantly enhancing overall process control including sample monodispersity.^[^ ^16^ ^]^ This technology offers efficient methods to produce, load, manipulate, and process highly monodisperse droplets, which are applicable in different fields such as soft materials, foods, biology, medicine, and chemistry.^[^ ^17^, ^18^ ^]^

Microfluidic methods have been frequently used for the generation of particle‐stabilized droplets and bubbles in recent years. Yet, challenges such as the high energy barriers that often prevent spontaneous adsorption of solid particles on the interface and clogging of microchannels caused by particles are still limiting the usage of particle‐stabilized emulsions and foams in microfluidics. This review provides a comprehensive overview of the fundamental principles of droplet generation and the materials used in microfluidic device fabrication, specifically in the context of Pickering emulsions and foams. We then delve into various particle loading methods, highlighting successful applications of microfluidics in particle‐stabilized emulsions and foams. Furthermore, we discuss the advantages and ongoing challenges that currently hinder the broader adoption of microfluidic techniques in this field. Finally, we offer our perspectives on the promising future applications of microfluidic approaches within this domain.

## 2 Microfluidics: Fundamentals and Fabrication Materials

### 2.1 Fundamentals of Droplet Generation

**Figure 1:**
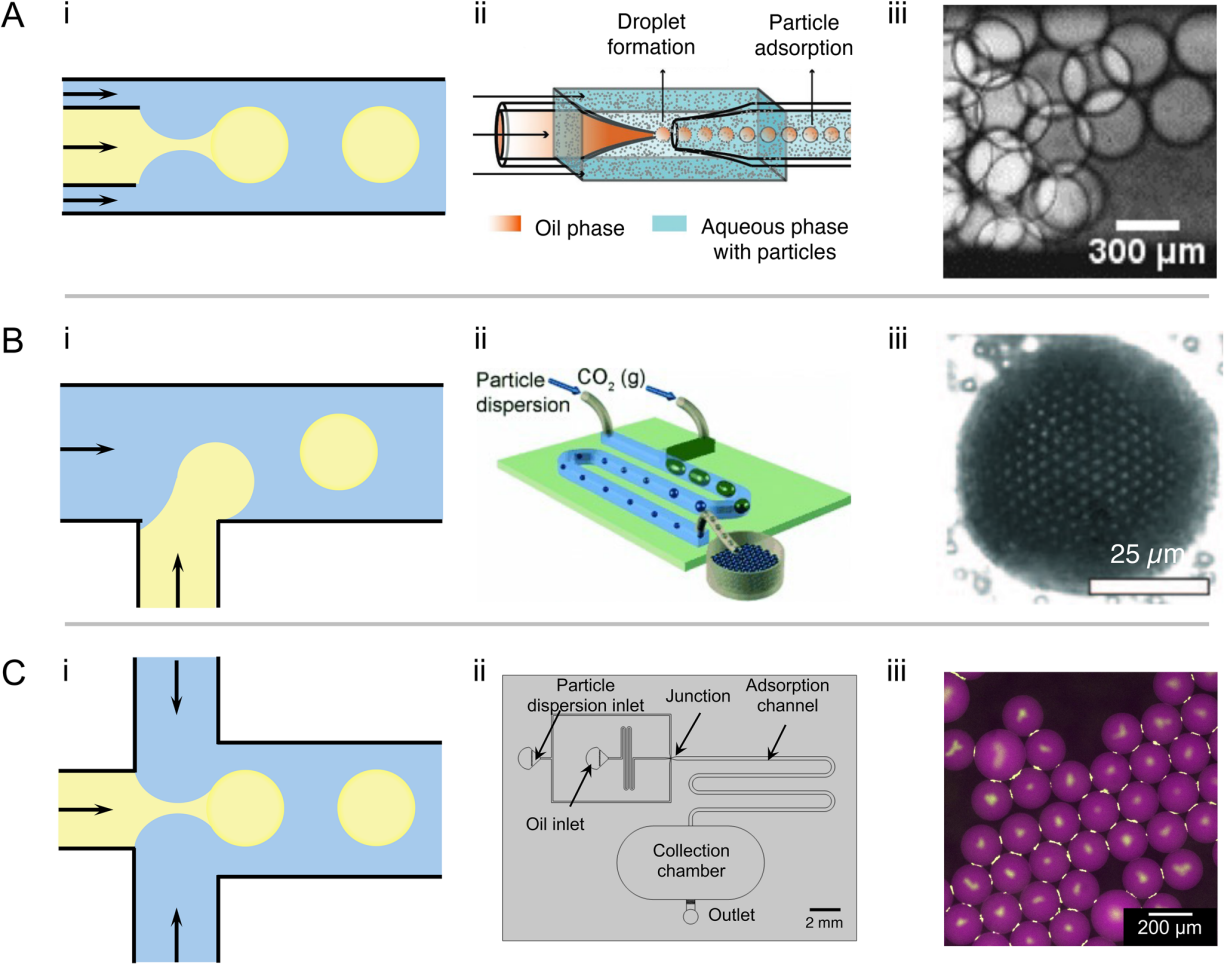
Microfluidic devices with various geometries used for generating particle‐stabilized droplets or bubbles. From left to right: (i) schematics of the droplet generation methods, (ii) schematics of the microfluidic devices, and (iii) the micrographs of the generated droplets or bubbles. A) A co‐flow method using a glass capillary device for droplet formation in dripping mode. Adapted with permission.^[^ ^24^ ^]^ Copyright 2018, Wiley‐VCH. B) A cross‐flow method using a PDMS device to generate armored CO_2_ bubbles. Reproduced with permission.^[^ ^25^ ^]^ Copyright 2009, Wiley‐VCH. C) A flow‐focusing method using a PDMS device to generate poorly covered Pickering droplets. Adapted with permission.^[^ ^13^ ^]^ Copyright 2025, Wiley‐VCH.^[^ ^13^, ^24^, ^25^ ^]^

In microfluidic devices, droplets are generated by transferring energy to the liquid–liquid interface; this energy can come directly from the hydrodynamic flow itself (passive control) or via an external input (active control).^[^ ^19^ ^]^ Depending on the type of external energy applied, active methods can be classified as mechanical, electrical, thermal, or magnetic.^[^ ^20^ ^]^ Although requiring additional energy input, they can provide more robustness and precision.^[^ ^21^ ^]^ While active control methods have not been applied for the generation of Pickering emulsions or foams as of yet, they are promising tools for future applications. **Figure** **1** shows the most common passive methods for droplet production, involving the use of three distinct geometries for droplet generation: co‐flow junction, in which immiscible fluids meet as parallel streams; cross‐flow junction, in which the immiscible fluid streams meet at an angle to one another; and flow‐focusing junction, in which there is a geometric element that causes the streams to accelerate, narrowing the inner fluid thread.^[^ ^22^ ^]^ In all these methods, droplet generation is achieved through shear. Moreover, one liquid can be dispersed into another by employing a spontaneous droplet formation method that relies on the Laplace pressure differences, such as terrace‐based systems.^[^ ^23^ ^]^

Droplet formation modes in microfluidics include squeezing, dripping, and jetting, determined by flow rates and fluid properties.^[^ ^26^ ^]^ The squeezing mode arises from channel confinement, producing “slug‐like” droplets. The dripping mode occurs with increased shear stress, where viscous forces dominate over interfacial tension effects. The jetting mode results from further increase in the shear stress, breaking an extended liquid jet into droplets. The transition to a stable jet occurs at even higher shear stress.^[^ ^27^ ^]^ Dimensionless parameters such as Reynolds number (Re = ρ*UL*/η (inertial force/viscous force)), Weber number (We = ρ*U* ^2^ *L*/γ (inertial force/interfacial tension force)), capillary number (Ca = *U*η/γ (viscous force/interfacial tension force)), Bond number (Bo = Δρ*gL* ^2^/γ (gravitational force/interfacial tension)), and the Péclet number (Pe = *UL*/*D* (advective transport rate/diffusive transport rate)) are commonly used to quantify the relative influence of forces in microfluidic devices, where ρ is the density, η is the dynamic viscosity, *U* is the characteristic velocity, *L* is the characteristic length scale, γ is the interfacial tension, and *D* is the diffusion coefficient. Among these dimensionless parameters, the capillary number is most often considered because it relates to droplet/bubble size. For Ca < 0.01, viscous stresses are insufficient to overcome the confinement imposed by the microchannel walls. In this regime, the droplet or the bubble size *L*_*db*_ scales linearly with the flow‐rate ratio of the inner phase and outer phase φ = *Q*_*i*_/*Q*_*o*_ and the channel width *w*,^[^ ^22^ ^]^ as *L*_*db*_/*w* = *A*_1_ + *A*_2_φ, where the coefficients *A*_1_ and *A*_2_ are obtained by fitting and vary with the cross‐sectional geometry of the pinch‐off region.^[^ ^28^ ^]^ At larger capillary numbers (Ca > 0.01), shear stresses become significant; the interface breaks less readily, and co‐flowing fluid threads form largely independent of the microfluidic geometry. The thread diameter *d*_*t*_ scales with the flow‐rate ratio according to *d*_*t*_ ∼ φ^1/2^.^[^ ^22^ ^]^ A detailed discussion of the above‐mentioned dimensionless parameters and their importance for microfluidic droplet generation is presented in a recent review work.^[^ ^29^ ^]^

### 2.2 Microfabrication Materials

Various materials can be utilized to produce microfluidic devices, including inorganic materials like silica, silicon and glass, organic materials like polydimethylsiloxane (PDMS), polytetrafluoroethylene (PTFE), and polystyrene (PS), and polymethyl methacrylate (PMMA), as well as hydrogel‐based and paper‐based materials.^[^ ^30^ ^]^ Nevertheless, the research on Pickering emulsions and foams primarily utilizes PDMS and glass capillary for the fabrication of microfluidic devices. PDMS excels at fast, low‐cost prototyping, integrated valves and pumps, and excellent optical properties, making it ideal for rapid method development. On the other hand, glass capillaries excel at axisymmetric 3D droplet generation, resistance to chemicals and pressure, and offer simple, reliable wetting control—especially for single‐ and double‐emulsions. Microfluidic devices fabricated from glass substrates with customized channel designs also show great potential, as they combine the advantages of both PDMS and glass capillary‐based devices.^[^ ^31^ ^]^

Soft lithography using elastomeric polymer molding has become one of the most important and popular methods for microfluidic device fabrication.^[^ ^32^ ^]^ By fabricating user‐defined microchannels within PDMS blocks and their subsequent bonding to glass slides, these devices can be used for making monodisperse emulsions and foams. Precise control of droplet size and breakup rate can be achieved by manipulating the channel geometry, fluid properties, and fluid flow rates. The silicon mold is reusable and can be replicated multiple times, facilitating rapid prototyping at low cost. Moreover, lithography enables the creation of highly complex flow channels for adjusting the fluid streams upstream and manipulating the droplets downstream, allowing multiple functions to be performed within a single device.

Despite these advantages of PDMS‐based microfluidic devices, there are some limitations in their application.^[^ ^33^ ^]^ One drawback is the interaction between the droplets and the channels, and the swelling of PDMS in the presence of some organic solvents.^[^ ^34^ ^]^ Additionally, PDMS can leach uncrosslinked oligomers from the curing process into the solution and absorb small molecules.^[^ ^35^ ^]^ Also, PDMS is vapor‐permeable, which can cause evaporation during long‐term experiments, thereby affecting the accuracy of the results.^[^ ^36^ ^]^

Capillary microfluidic devices have also been extensively investigated for the production of uniform emulsions and foams.^[^ ^37^ ^]^ Typically, the device comprises coaxially arranged glass capillaries fixed on glass slides. One fluid is directed through the inner capillary while another fluid is directed through the outer capillary, either in the same or in the opposite direction. This configuration results in the formation of highly monodisperse emulsions and foams. Glass capillary microfluidic devices offer an inherent advantage in that their wettability can be controlled through surface modification using appropriate surface modifiers, thereby eliminating solvent compatibility issues. Although capillary‐based microfluidics offer versatility in droplet generation, they do have several drawbacks. Firstly, the invariable geometry of glass capillaries in modular assembly does not allow for the construction of precise and complicated microfluidic systems.^[^ ^38^ ^]^ Additionally, the droplet size is generally determined by the tip size in capillary microfluidics, with prepared droplets typically ranging from 100 µm and above. Smaller tips are required to produce smaller droplets; however, they are prone to clogging and are challenging to fabricate.^[^ ^37^ ^]^

Unlike the assembly of capillaries, microchannels can also be directly fabricated on glass substrates using wet/dry etching, machining, or laser cutting.^[^ ^39^ ^]^ These glass‐based microfluidic devices have also been used in the study of Pickering emulsions. Glass‐based microfluidic devices offer several advantages, making it a preferred material in specific cases. Glass is highly resistant to most chemicals, including organic solvents, acids, and bases, making it ideal for applications involving aggressive chemicals or reactive compounds that could otherwise degrade polymeric materials such as PDMS. Additionally, glass is mechanically strong and less prone to deformation over time compared to soft materials like PDMS, ensuring device longevity and reproducibility in high‐pressure systems. Besides, for mechanistic investigations, precise knowledge of channel dimensions is essential, which is significantly more feasible with glass due to its structural stability and minimal dimensional variability compared to PDMS. Moreover, unlike PDMS, glass is impermeable to gases, making it suitable for applications requiring a controlled gas environment or the prevention of gas exchange. However, some disadvantages limit the use of glass‐based microfluidics, including higher costs due to the complexity of processing (e.g., etching and bonding), the requirement for cleanroom facilities, challenges during fabrication, and brittleness during use.^[^ ^40^ ^]^ In addition to the three materials mentioned above, microfluidic devices made of silica capillaries,^[^ ^41^, ^42^ ^]^ silicon,^[^ ^43^ ^]^ or Teflon^[^ ^44^ ^]^ have also been used in the study of Pickering emulsions. However, due to the very limited use of such materials, we will not discus them in detail in this review.

## 3 Microfluidic Production of Particle‐Stabilized Emulsions and Foams

Creating Pickering emulsions and foams in microfluidic channels is challenging due to the weak nature of the hydrodynamic forces present in microchannels, which are often insufficient for particles to surpass the adsorption energy barrier.^[^ ^13^ ^]^ Therefore, addressing particle adsorption is crucial for applying microfluidics in the study of particle‐stabilized emulsions and foams. Several approaches of loading particles onto fluid interfaces have been effectively employed to generate particle‐stabilized droplets and bubbles, which can be classified as: particles loading from the continuous phase, particles loading from the dispersed phase, and particles initially dispersed in the middle phase of a double emulsion droplet (a droplet encapsulated within a larger droplet). The published literature on the use of microfluidics to study Pickering emulsions and foams is summarized in **Table** **1**.

**Table 1:** Particle‐stabilized droplets and bubbles prepared using microfluidics

Device material and principle	Particle			Emulsion or foam type	Remarks	Particle loading method	Refs.
	Material	Diameter [um]	Concentration [wt%]				
PDMS: Flow‐focusing	PS; PMMA; Silica;Gold	1.0–6.0	0.1	Mineral oil or Air/Water		○	[45, 46]
	P(DVB‐MAA)	3.5	8	Water‐Ethanol/Hexadecane		•	[47]
	P(DVB‐MAA)	3.5	14	DMPA‐TPGDA/Water	⋆	•	[47]
	Silica	0.5–1.0	3.0–3.4	Water/PGD		•	[48]
	P(S‐AA)	2.8	1.2	CO_2_/Water‐Glycerol	⋆	○	[49]
	Fluorinated silica	0.05–5.0	0.5–5.0	Water/Fluorinated oil		○	[50]
	Fluorinated silica	0.1	1.5	Water/Fluorinated oil		○	[51]
	PS	0.5–4.5	0.5	Air/Water	⋄	○	[52]
	PNIPAM	0.364–0.805	0.4–0.8 *	Benzyl benzoate/Water		○	[53]
	PS‐PVP	0.45–2.48 *	4.0	Air/Water		○	[54]
	Fluorinated silica	0.03–0.2	6	Water/Fluorinated oil		○	[55]
	PS; PAA	0.1	0.6	Dextran‐Water/PEG‐Water		•	[56]
	PS	1.0–10.0	0.3–14.9	Dextran‐Water/PEG‐Water		○	[57]
	Fluorinated silica	0.065	0.1–1.2	Water/Fluorinated oil		○	[58]
	CM‐AHPA	0.07	0.1	Water/Octanol		•	[59]
	CMp‐vTA	0.3	0.1	Octanol/Water		•	[59]
	CMp‐vTA	0.3	0.1	Water‐Styrene‐Octanol/Water		•	[59]
	Janus CP(S‐AA)‐PS	0.272–0.45 *	2.9	Water/Hexadecane		•	[60]
	Janus CP(S‐AA)‐PS	0.272–0.45 *	2.2	Hexadecane/Water		•	[60]
	Janus PS‐Fe_3_O_4_ · SiO_2_	0.15 *	0.2	Water/Hexadecane		•	[61]
	Zein nanoparticle; HPMC	unknown	3; 0.5–3.0	Soybean oil/Water		○	[62]
	PS	2	0.0625–0.25	Dodecane/Water		○	[13]
PDMS: Cross‐flow	Silica; P(S‐AA); P(VP‐MMA)	0.7–3.0	1.5	N_2_ or CO_2_/Water	⋆	•	[25]
	Silica	0.012	2	N_2_/Water		○	[63]
Glass capillary: Co‐flow	Silica	0.111	10–40 *	HDODA‐ETPTA ‐HMPP‐Toluene/Water	⊗	○	[64]
	Silica	0.01	7.5		⊗	⊚	[65]
	Silica	0.205‐0.9	10	Water‐ETPTA‐Photoinitiator/Water	⊗	⊚	[66]
	Silica	0.23	10	Water‐ETPTA‐Photoinitiator/Water	⊗	⊚	[67]
	Silica	0.205–0.9	10	Water‐ETPTA‐Photoinitiator/Water	⊗	⊚	[68]
	PMMA	0.01	0.1–5.0	Air/Glycerol		○	[69]
	PS	0.1–0.3	0.0625–1.0	Dodecane/Water	⋄	○	[24]
	Gelatinized starch	unknown	4.0	Water/Sunflower oil/Water	⋄, ⊗	○	[70]
	Silica	0.12	0.04	Water/Dichloromethane/Water	⊗	•	[71]
	Janus PS‐Fe_3_O_4_ · SiO_2_	0.15 *	0.2	Hexadecane/Water		○	[61]
Glass capillary: Flow‐focusing	Silica; PS	0.111–0.4	10.5–22.0 *	HDODA‐HMPP/Water	⊗	○	[72]
	TEG‐Au; CdSe QDs	unknown; 0.006	0.2–0.5	Water‐TCB/Water		○	[73]
	Ethyl cellulose	0.1–0.3	1	Air/Water		○	[74]

Device material and principle	Particle			Emulsion or foam type	Additional information	Particle loading method	Refs.
	Material	Diameter (um)	Concentration (wt%)				
Glass: Flow‐focusing	Silica	0.01	0.1–0.5	Dodecane/Water		○	[75]
	P(NIPAM‐MA) micogel	0.27	0.8	Decane/Water	⋄	○	[76]
	Silica	0.6	0.3–4.8	Fluorinated oil/Water‐Ethylacetate‐ Ethanol		⊚	[77]
	Hollow silica	1.9	8.9	1‐butanol‐n‐hexadecane/Water		⊚	[77]
	Silica	0.6	unknown	Water‐Glycerol/Decanol		⊚	[77]
Glass: Cross‐flow	Phyllosilicate	0.03 *	0.5–1.0	Paraffin/Water	⋄	○	[78]
	Silica	0.02–0.03	0.2–0.4	Water/Silicone oil	⊗	•	[79]
	Lipid	0.17	5	Sunflower oil/Water		○	[80]
Silica capillary: Cross‐flow	PNIPAM; P(NIPAM‐AA) microgel	0.273–1.02	0.001–0.5	Air or Dodecane/Water	⋄	○	[41]
	Fluorinated silica	0.1	3	Water/Fluorinated oil	⊗	○	[42]
Silicon and Glass: Cross‐flow	Silica	0.1–0.5	0.4	Tricaprylin/Water		○	[81]
Silica and Teflon: Co‐flow	Au; Silica	0.0165; 0.165	8.3 *; 0.05	Water (Au)/Cyclohexane (Silica)	⋄	•, ○	[44]
	Fe_3_O_4_/SiO_2_	0.4	unknown	ETPTA/Water		○	[82]
Silica and Epoxy resin: Co‐flow	Bovine serum albumin‐pepsin	unknown	0.44	Water/Water		○	[83]
Unknown: Flow‐focusing	Silica	0.012	1	Oil/Water	⊗	○	[84]

Name	Abbreviation	Name	Abbreviation
1,6‐hexanediol diacrylate	HDODA	poly(4‐vinylphenol‐co‐methyl methacrylate)	P(VP‐MMA)
2,2‐dimethoxy‐2‐phenylacetophenone	DMPA	propylene glycol diester	PGD
2‐hydroxy‐2‐methyl‐1‐phenyl‐1‐propanone	HMPP	poly(styrene‐co‐acrylic acid)	P(S‐AA)
3‐allyloxy‐2‐hydroxy‐1‐propanesulfonic acid	AHPA	polyacrylic acid	PAA
4‐methyl‐5‐vinylthiazole	vTA	polymethyl methacrylate	PMMA
2,4‐diamino‐6‐phenyl‐1,3,5 triazine	CMp	polyethylene glycol	PEG
cyanuric acid‐melamine	CM	poly(N‐isopropylacrylamide)	PNIPAM
cadmium selenide quantum dots	CdSe QDs	poly(N‐isopropylacrylamide‐co‐acrylic acid)	P(NIPAM‐AA)
cross‐linked poly(styrene‐co‐acrylic acid)‐polystyrene	CP(S‐AA)‐PS	poly(N‐isopropylacrylamide‐co‐methacrylic acid)	P(NIPAM‐MA)
ethoxylated trimethylolpropane triacrylate	ETPTA	tripropylene glycol diacrylate	TPGDA
polystyrene	PS	trichlorobenzene	TCB
polyamide	PA	tetra(ethylene glycol)	TEG
poly(divinylbenzene‐methacrylic acid)	P(DVB‐MAA)	polystyrene‐polyvinylpyrrolidone	PS‐PVP
hydroxypropyl methylcellulose	HPMC		
Name	Symbol	Name	Symbol
Salt added	⋄	Surfactant added	⊗
NaOH added	⋆	Particles loading from the outer phase	○
Particles loading from the inner phase	•	Particles loading from the middle phase	⊚
Estimated values	*		

### 3.1 Particle Loading from the Continuous Phase

**Figure 2:**
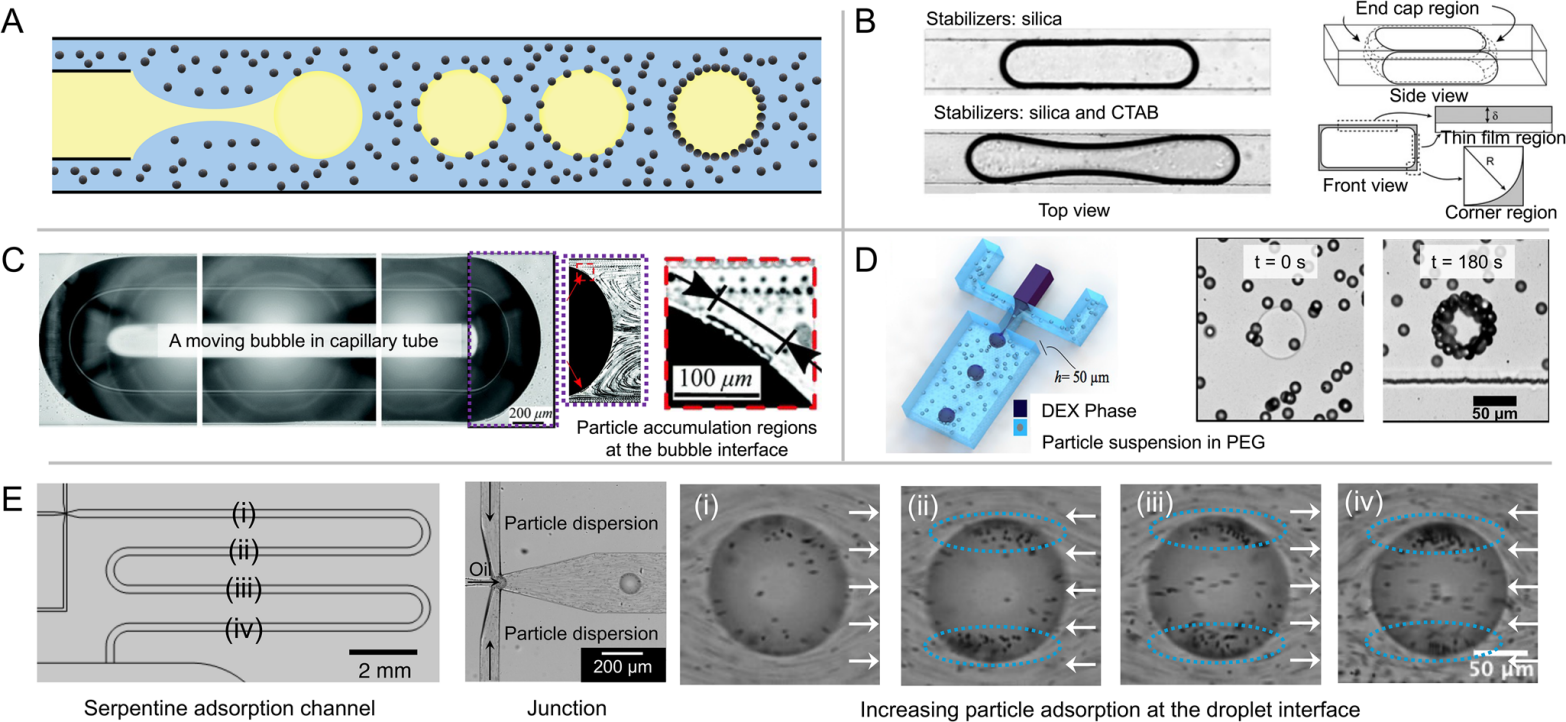
Formation of Pickering emulsions and foams via particle loading from the continuous phase. A) Schematic of particle loading from the continuous phase. B) Particles adsorb to the interface from the rear of the bubble, the thin film region along the channel, or the open region in the microchannel corners as the bubble moves through a rectangular capillary tube. Bubbles generated in the continuous phase containing only silica particles exhibit different shapes compared to those formed with silica particles and CTAB surfactants. Adapted with permission.^[^ ^63^ ^]^ Copyright 2012, Royal Society of Chemistry.C) Front, middle and rear segments of a long bubble translating in a circular capillary tube with PMMA particle dispersion. Particles accumulate and adsorb from the rear of the bubble. Adapted with permission.^[^ ^69^ ^]^ Copyright 2017, Royal Society of Chemistry. D) Particle loading from the continuous phase to a water‐water interface in a PDMS flow‐focusing microfluidic device and time‐series images of the 12.8 w/v % dextran (DEX) droplets in 10 w/v % polyethylene glycol (PEG) being covered with carboxylated polystyrene particles. Adapted with permission.^[^ ^57^ ^]^ Copyright 2018, American Chemical Society. E) Particle loading from the continuous phase to an oil‐water interface in a PDMS flow‐focusing microfludic device. Particles adsorb as the droplet flows down the adsorption channel and show the asymmetric accumulation at the droplet interface. Adapted with permission.^[^ ^13^ ^]^ Copyright 2025, Wiley‐VCH.^[^ ^13^, ^57^, ^63^, ^69^ ^]^

Among the existing methods for loading particles onto droplet interfaces, introducing particles from the continuous phase has found the broadest application, as schematically illustrated in **Figure** **2**A. When dispersing particles in the continuous phase, two main approaches have been utilized with respect to the timing of particle adsorption onto the droplets: before and after the droplet break‐off at the junction. Subramaniam et al.^[^ ^45^ ^]^ were the first to propose a microfluidic method for preparing particle‐stabilized droplets and bubbles using a flow‐focusing microfluidic device. This approach delivers colloidal particles to the droplet interface via hydrodynamic flows before the droplets break off, resulting in the formation of particle‐coated droplets and bubbles. There is always an adsorption barrier for a particle to breach the interface due to the image‐charge repulsive force^[^ ^85^, ^86^ ^]^ and the hydrodynamic thinning of the liquid film between the particle and the interface.^[^ ^87^ ^]^ As a result, the adsorption of particles is a non‐spontaneous process, requiring energy input through hydrodynamic flows to overcome this barrier. Despite the initial promise of this method, further research has been limited, possibly due to the need for precise control of particle adsorption before droplet formation and the relatively slow rate of droplet production.

An alternative method is loading the particles after the formation of the droplet. In microfluidic systems, the transport mechanisms of particles from bulk to interface are significantly influenced by the interplay between convection and diffusion, quantitatively described by the Péclet number. A large Pe > 1 indicates convection‐dominated transport, while a small Pe < 1 signifies diffusion‐dominated transport. Yao et al. suggested that the particle adsorption at the interface is diffusion‐limited,^[^ ^24^ ^]^ even when the calculated Pe was ≫ 1 given the experimental conditions. It was observed that due to the laminar nature of the Stokes flow, particles tend to travel smoothly around the droplet,^[^ ^13^ ^]^ which means the final step of the adsorption should always be diffusion‐limited. For particle radii between 1 nm and 1 µm in water at room temperature, with a fluid velocity of 1 mm/s and a characteristic length of 100 µm, Pe ranges from 4.08 × 10^2^ to 4.08 × 10^5^, demonstrating the significant influence of advection even for the smallest particles. Despite convection transporting most particles from the bulk continuous phase close to the droplet surface,^[^ ^55^ ^]^ effective particle loading remains challenging because laminar flow conditions in microfluidics often lack sufficient force to overcome the energy barrier at the oil–water interface.^[^ ^76^ ^]^ The energy barrier *E*_*a*_ for particles to penetrate the interface determines the adsorption rate as described by an Eyring‐type equation based on transition state theory, as outlined by Kramers:^[^ ^2^, ^88^ ^]^ $k_{ad}=\omega_{0}\cdot e^{-E_{a}/RT}$, where *k*_*ad*_ represents the adsorption rate and ω_0_ is the attempt frequency associated with particle diffusion. Under laminar flow conditions, the drag force acting on a particle can be estimated using Stokes' law, *F* = 6πη*r*_*p*_ *v*, where η is fluid viscosity, *r*_*p*_ the particle radius, and *v* the relative fluid velocity. Then the drag force can be decomposed in a contribution perpendicular to the interface, *F*_⊥_, which pushes the particle against the interface. These hydrodynamic forces influence adsorption, as expressed in: $k_{ad}=\omega_{0}\cdot e^{(-E_{a}+F_{⊥}\delta )/RT}$, where δ represents the thickness of the interfacial region that must be broken for adsorption to occur.^[^ ^2^, ^89^ ^]^ By lowering the energy barrier, hydrodynamic forces significantly enhance the adsorption rate with increasing flow velocity, ultimately promoting stability. Experimental results from producing lipid particle–stabilized droplets using a microfluidic device have shown that larger fluid flow enhances particle adsorption, leading to exponentially increasing adsorption rates and ultimately greater surface coverage, in agreement with model predictions.^[^ ^80^ ^]^ Kotula and Anna reported that when confined droplets or bubbles translate inside capillary tubes, particles can adsorb to interfaces in three distinct regions: the curved end caps, the thin film along channel walls (in rectangular channels), and the open fluid flow regions in microchannel corners,^[^ ^63^ ^]^ as shown in Figure 2B. The lubrication film between drops and channel walls enhances particle loading through increased convective effects.^[^ ^63^ ^]^ Yu et al.^[^ ^69^ ^]^ observed particle accumulation at the rear of bubbles moving in circular capillary tubes (Figure 2C), attributing adsorption to a sliding mechanism where particles approach the interface, draining the intervening fluid and adhering due to van der Waals forces.

In many instances, Pickering emulsions or foams are prepared using a combination of particles and various additives that enhance particle adsorption at interfaces.^[^ ^90^ ^]^ The addition of salts to water is a common approach, as salts can screen particle charges and reduce electrostatic repulsive force between particles and interfaces. However, it should be noted that the addition of salt can also screen the charges at the hydrophobic/water interface,^[^ ^91^ ^]^ thereby reducing electrostatic repulsion and promoting contact between two bare interfaces, which initiates coalescence. For charged microgels, increasing their concentration (thus increasing endogenous ions) can also provide self‐screening effects.^[^ ^41^ ^]^ Adjusting the pH of the aqueous phase can alter the charge on the particles with ionizable surface groups like –SiOH, –COOH, or –NH_2_, thereby influencing their adsorption. The adsorption of oppositely charged surfactants can hydrophobize particle surfaces, enhancing their activity at the oil‐water interface.^[^ ^63^, ^64^ ^]^ However, with such a mixed interface where both surfactants and particles are adsorbed, these systems cannot be considered to be true Pickering emulsions, although most of the stabilizing effect can still be the result of particles being present.^[^ ^2^ ^]^ Fluorinated silica nanoparticles exhibit amphiphilic properties when dispersed in fluorinated oils and can be effectively used to prepare particle‐stabilized emulsions within microfluidic devices.^[^ ^42^, ^50^, ^51^, ^55^ ^]^ Notably, these nanoparticles can spontaneously adsorb to the water‐fluorinated oil interface without the need of an external flow due to their amphiphilic structure.^[^ ^55^ ^]^ When the dielectric constants of the dispersed and continuous phases are similar, as in aqueous two‐phase systems, the resulting negligible electrostatic repulsion further promotes particle adsorption, shown in Figure 2D.^[^ ^57^ ^]^ Additionally, higher particle concentrations and longer channel lengths can increase the particle coverage at droplet or bubble interfaces, promoting stabilization. However, a higher particle concentration concomitantly increases the risk of channel clogging.^[^ ^72^ ^]^ Therefore, a serpentine adsorption channel, as shown in Figure 2E, is recommended for use. Our recent work clearly demonstrates the gradual adsorption process of particles onto the droplet interface as the droplet flows through a serpentine adsorption channel.^[^ ^13^ ^]^

### 3.2 Particles Loading from the Dispersed Phase

**Figure 3:**
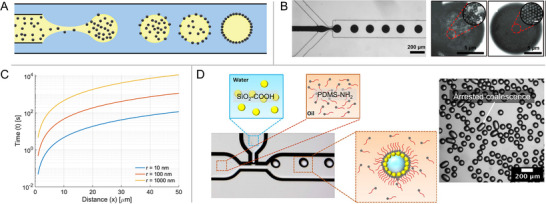
Pickering emulsion production via particle loading from the dispersed phase. A) Schematic of particle loading from the dispersed phase. B) Particle loading from the dispersed phase to an oil‐water interface in a PDMS flow‐focusing microfluidic device and the corresponding confocal microscopy images of the water‐ethanol droplets in hexadecane armored with a shell of poly(divinylbenzene‐methacrylic acid) (poly(DVB‐MAA)) particles. Adapted with permission.^[^ ^47^ ^]^ Copyright 2008, American Chemical Society. C) The diffusion time (*t*) for particles to travel from the center of a droplet to the interface is presented as a function of distance (*x*), for particles with radii of 10 nm, 100 nm, and 1000 nm, at 20 °C in a medium with a viscosity of 1 mPa s. The diffusion time was calculated using the Einstein‐Smoluchowski relation, demonstrating the quadratic dependence on distance and the direct proportionality to particle radius. D) Micrograph of the microfluidic droplet generation in a T‐junction geometry. The water phase has acid‐treated silica nanoparticles, and the amine‐terminated polymer ligands are dissolved in the oil phase. Adapted with permission.^[^ ^79^ ^]^ Copyright 2018, American Chemical Society.^[^ ^47^, ^79^ ^]^

**Figure** **3**A shows the schematic of producing Pickering emulsions by loading the particles from the dispersed phase. In this case, convection‐ as well as diffusion‐induced adsorption occurs within the droplet. In microfluidic systems, the internal flow velocity is typically lower than the external flow velocity, particularly in highly viscous droplets.^[^ ^92^, ^93^ ^]^ This discrepancy arises because the internal flow is primarily driven by shear forces transmitted through the droplet's interface from the external fluid, leading to slower internal movement. Moreover, internal flow often manifests as recirculating or vortex flows, further reducing its velocity. These findings suggest that the effect of diffusion on particle adsorption within the droplet must be considered and has a dominant role.

The pioneering work on dispersing particles within droplets in microfluidics, referred to as the ‘Inside‐Out’ method, was published by Nie et al.^[^ ^47^ ^]^ This method was successfully employed in the production of both oil‐in‐water and water‐in‐oil Pickering emulsions, as shown in Figure 3B. Once the droplets are formed, there is a finite time required for the interface to become fully populated with particles. This time scale is determined by the diffusion of particles within the confined fluid as they undergo Brownian motion, as well as the droplet size. An upper‐bound estimate of the time required for the particles to diffuse to the interface can be obtained by the equation $t\approx \frac{x^{2}}{D}$, where *x* represents the distance, essentially the droplet size, over which the particle must diffuse to approach the surface, while *D* represents the diffusion coefficient. The diffusion time is typically on the order of several seconds for particles with a diameter of approximately 1 µm to reach the interface of a droplet with a radius of around 60 µm,^[^ ^48^ ^]^ which is comparable to the time required to load particles from the continuous phase.^[^ ^24^, ^63^ ^]^ In Figure 3C, we calculate the diffusion time as a function of distance for particles with radii of 10, 100, and 1000 nm at 20 °C in a medium with a viscosity of 1 mPa s, the results show that for sub‐micron radii particles, a few seconds are sufficient to diffuse to the interface for typical droplet diameter around 100 µm. However, this can be a concern for high‐throughput production, as few seconds are still significantly longer than the time required for droplet formation and coalescence.^[^ ^94^ ^]^

Additives have also been utilized to enhance the migration of particles to the droplet interface. Both PEG and dextran can adsorb onto polystyrene particles, thereby altering the contact angle and further reducing the interfacial free energy, which results in the surfactant‐like, spontaneous interfacial adsorption of amine‐modified polystyrene particles at the water/water interface.^[^ ^56^ ^]^ Janus particles with tunable amphiphilicity enable adsorption onto the interface in a manner similar to conventional surfactants.^[^ ^60^ ^]^ By adding PDMS‐NH_2_ to the continuous phase, negatively charged nanoparticles diffuse to the interface and interact with the protonated amines, forming particle‐surfactant complexes,^[^ ^79^ ^]^ as shown in Figure 3D. Lastly, as higher particle concentrations in the continuous phase tend to cause microchannel clogging, their concentration is usually kept below 1 wt%. In contrast, higher concentrations (3 wt%) can be used when loading the droplet interior, which can be of advantage.

### 3.3 Particle Loading from the Middle Phase of Double Emulsions

**Figure 4:**
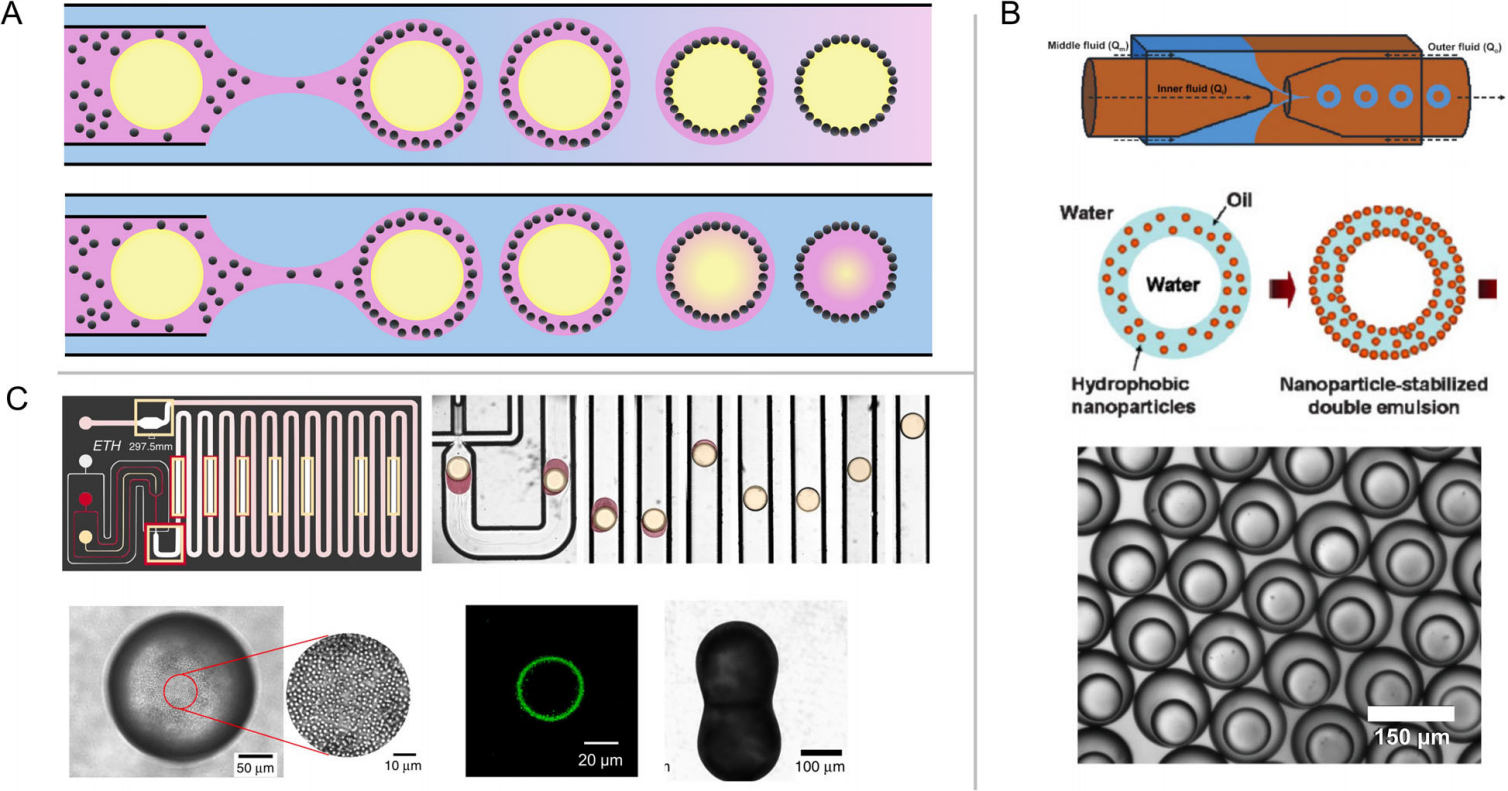
Particle loading from the middle phase of double emulsion droplets. A) Schematic illustrating particle loading from the middle phase of a double emulsion droplet. During droplet flow in the channel, the middle phase dissolves into either the outer phase or the inner phase. B) From top to bottom: schematic representation of the microfluidic device for double emulsion generation; schematic showing the formation of particle‐stabilized water‐in‐oil‐in‐water double emulsions; optical microscope images of the collected double emulsions. Adapted with permission.^[^ ^65^ ^]^ Copyright 2008, Wiley‐VCH. C) Top panel, from left to right: Schematic illustration of the microfluidic chip showing the inlets for the outer (white), middle (red), and inner (beige) phases. Transient double emulsion droplets captured at various positions along the channel. Bottom panel, from left to right: Optical micrographs and a confocal micrograph depicting silica particle adsorption at the interface and arrested coalescence of the prepared droplets. Adapted with permission.^[^ ^77^ ^]^ Copyright 2018, Springer Nature.^[^ ^65^, ^77^ ^]^

The third method involves a transient double emulsion, in which the particles are dispersed in the middle phase of a double emulsion droplet, as schematically shown in **Figure** **4**A. This method was first introduced by Lee and Weitz,^[^ ^65^ ^]^ using glass microcapillary devices to generate water‐in‐oil‐in‐water double emulsions with polyvinyl alcohol (PVA) added to both the aqueous phases. The particles initially dispersed in the middle oil phase adsorb to the two oil/water interfaces, thereby stabilizing the droplets, as shown in Figure 4B. Microcapsules with shells composed of colloidal particles, also known as colloidosomes, are formed after the prepared double emulsions are exposed to a vacuum for a sufficient period. Kim and co‐authors utilized microcapillary devices equipped with a UV exposure unit to prepare photocurable water‐in‐oil‐in‐water double emulsion droplets, where the middle phase consisted of a photocurable resin with silica particles.^[^ ^66^, ^68^ ^]^ This method enables the fabrication of functional particles by employing silica particle arrays on the surfaces of the droplets as efficient binding sites for immobilizing target biomolecules through precise surface chemistry. When the compositions and phase volume ratios are selected such that the middle phase completely dissolves into either the inner or outer phase, particle‐stabilized single emulsions can be formed via transient double emulsions within microfluidics, without the need for additives, as shown in Figure 4C.^[^ ^77^ ^]^ This approach provides a versatile method for controlling the surface coverage and composition of droplet interfaces.

## 4 Advantages and Challenges of Microfluidics in Pickering Emulsions and Foams

Microfluidic systems offer a controllable platform for gaining valuable insights into the fundamental mechanisms underlying Pickering emulsions and foams. They offer several advantages for the production and investigation of Pickering emulsions and foams, and we briefly explain them below:

(*i*) The droplets generated via microfluidics, when the key parameters such as flow rate and channel dimensions are kept constant, experience similar shear and flow conditions and thus have comparable particle coverage, which aids to perform subsequent quantitative analysis and statistically relevant experimentation.^[^ ^76^ ^]^ Microfluidic devices enable precise control over droplet size, typically achieving a coefficient of variation below 5%, and thus generating droplets with a uniform size distribution. The monodispersity of droplets or bubbles can also significantly reduce Ostwald ripening by minimizing the effective Laplace pressure differences due to their uniform size.^[^ ^95^ ^]^
(*ii*) High mechanical shear used to produce smaller droplets during conventional emulsification methods can also destabilize or deform fragile particles or aggregates during the emulsification, which can be avoided by microfluidic emulsification.^[^ ^43^ ^]^
(*iii*) In contrast to the conventional homogenization emulsification methods, where even less than 1–5% of the energy is used for emulsification and the remaining is dissipated as heat,^[^ ^96^ ^]^ microfluidics offer a low‐energy emulsification method. Moreover, the lack of heat generation prevents possible destabilization of thermo‐sensitive particles and liquids.^[^ ^11^ ^]^
(*iv*) Microfluidics can deal with very small volumes of fluids, down to 10^−9^ to 10^−18^ liters, making it possible to work with very small amounts of sample and applications requiring precision and efficiency in small‐scale fluid handling.^[^ ^97^ ^]^ In the development of functional particles, microfluidics can be employed to evaluate particle functionality, thereby accelerating the design of tailored particles for specific applications.
(*v*) Microfluidic platform enables the integration of magnetic, electric, and optical fields, providing a versatile system for studying multiphysics phenomena in a controlled environment.^[^ ^98^ ^]^
(*vi*) Microfluidics offers an excellent experimental platform for the in situ observation of the particle adsorption process at both droplet‐ and even particle‐level.^[^ ^45^ ^]^ Furthermore, the integrability of microfluidics allows us to collect, monitor, and manipulate samples within the same device.^[^ ^17^ ^]^

It is worth noting that the current research on Pickering emulsions and foams using microfluidics is primarily focused on harnessing the benefits of microfluidics in terms of droplet size and homogeneity control. Thus, numerous other advantages remain largely unexplored and remain an excellent avenue for further innovation. Nevertheless, there are also challenges in using microfluidics to prepare Pickering emulsions and foams, which we outline below, along with possible solutions:

(*i*) Microfluidic devices typically operate under laminar conditions, which hardly provide the particles with the necessary hydrodynamic forces to overcome the energy barrier for breaching the oil–water interface.^[^ ^45^, ^76^ ^]^ Although the adsorption of particles is thermodynamically favored, experimental studies demonstrate that the process is neither spontaneous nor rapid for colloidal particles. This is primarily because the majority of particles carry a charge to prevent colloidal aggregation and charged particles approaching a fluid‐fluid interface can experience electrostatic and image charge repulsion forces, which prevent the particles from breaching the interface.^[^ ^85^, ^99^ ^]^ Electrostatic energy barriers can be suppressed through the addition of salt which screens the surface charges; however, this may also lead to coagulation. Thus, sufficient hydrodynamic force is still necessary to enhance the particle adsorption rates.^[^ ^100^ ^]^
(*ii*) While a single junction can generate thousands of droplets per second, the volume throughput at this rate remains in the range of tens of kilograms per year, which is far from the industrial‐scale production necessitating the production of tons of emulsions.^[^ ^96^ ^]^ The edge based droplet generation (EDGE) technology,^[^ ^101^, ^102^, ^103^, ^104^ ^]^ enables simultaneous formation of multiple droplets within a single unit through spontaneous droplet generation. A key feature of this design is that the droplets form along the entire length of the plateau, with droplet size scaling proportionally to the plateau height by a factor of six. Additionally, it has been demonstrated that several hundred droplet formation units can be operated on a single chip, all supplied by a single feed channel, creating opportunities for large‐scale parallelization of microfluidic devices.
(*iii*) The high concentration of dispersed particles can result in the clogging of channels by aggregation and/or adhesion of particles to the microchannel surfaces, leading to unstable or uncontrollable flow and droplet generation.^[^ ^105^ ^]^
(*iv*) When using microfluidic devices made of PDMS, the inherent hydrophobicity of PDMS will prevent the formation of either emulsions or foams with water as continuous phase. In order to enable such emulsion formation, the continuous aqueous channel in the microfluidic device should be rendered hydrophilic after preparation. Oxygen plasma treatment is the most common method to hydrophilize PDMS surfaces because it is fast, benign, and effective.^[^ ^106^ ^]^ However, a serious drawback of this approach is that the effect of plasma treatment is temporary, and thus not desirable in long‐term applications.^[^ ^107^ ^]^ In the application of particle‐stabilized droplets or bubbles, several surface treatment methods have been used to improve the long‐lasting hydrophilicity of PDMS microfluidic chips, including coating the channels^[^ ^59^, ^60^ ^]^ or immediately filling the channels with distilled water after plasma bonding.^[^ ^52^, ^63^ ^]^ Our recent research shows that plasma treatment at a power of 18 W for 60 s makes the surface sufficiently hydrophilic. Immediately followed by filling the device with MilliQ water renders the surface sufficiently hydrophilic for at least 4 weeks.^[^ ^13^ ^]^ It should be noted that although hydrophilic treatment of surfaces with surfactants such as polyvinyl alcohol is widely applied,^[^ ^108^, ^109^, ^110^ ^]^ such surface treatments need to be carefully considered when producing Pickering emulsions and foams, as surfactants will affect the stabilizing mechanism of the particles.
(*v*) The mechanisms of surfactants promoting droplet breakup and preventing droplet coalescence have been well studied.^[^ ^111^ ^]^ On the other hand, the factors controlling droplet or bubble generation are significantly different in particle‐stabilized systems.^[^ ^75^ ^]^ For instance, the pinch‐off of particle‐laden interfaces,^[^ ^112^ ^]^ the distribution of stabilizers at droplet interfaces,^[^ ^13^ ^]^ and the movement of particle‐laden droplets inside microchannels^[^ ^113^ ^]^ all differ from those in surfactant‐stabilized systems. Overall, the mechanisms governing the dynamics of particle‐stabilized droplets or bubbles in microfluidic devices remain poorly understood.

## 5 Toward Applications of Microfluidics in Pickering Emulsions and Foams

The advantages of microfluidics—such as serving as templates for the synthesis of functional particles, enabling efficient encapsulation, and facilitating on‐chip analysis—combined with the unique properties of Pickering emulsions and foams, make them highly promising for a broad range of applications, including food, medical, and petroleum industries. This section discusses recent advancements in microfluidic technologies, with a focus on Pickering emulsions stabilized by Janus particles, encapsulation strategies for diverse applications, on‐chip analytical techniques, and the scaling‐up of microfluidic platforms for commercial use.

### 5.1 Pickering Emulsions with Janus Particles

There is a growing interest in the use of multi‐compartment particles due to their wide range of potential applications. Janus particles interact anisotropically with surrounding phases, provide Pickering stabilization at oil–water interfaces through their amphiphilicity, and are capable of simultaneously loading and transporting various substances.^[^ ^114^ ^]^ Pickering emulsions produced via microfluidic devices can serve as templates for preparing Janus particles.^[^ ^82^ ^]^ These Janus particles consist of amphiphilic magnetic mesoporous silica nanoparticles coated with polydopamine and platinum, can effectively remove pollutants such as dyes from water, demonstrating potential for wastewater treatment. The controlled self‐assembly of Janus nanoparticles at droplet interfaces allows droplets to move via thermophoresis, catalytic reactions, and magnetic fields. In addition to on‐chip preparation and the use of Janus particles as Pickering stabilizers, particles prepared off‐chip have also been employed to produce Pickering emulsions in microfluidic devices.^[^ ^61^ ^]^

**Figure 5:**
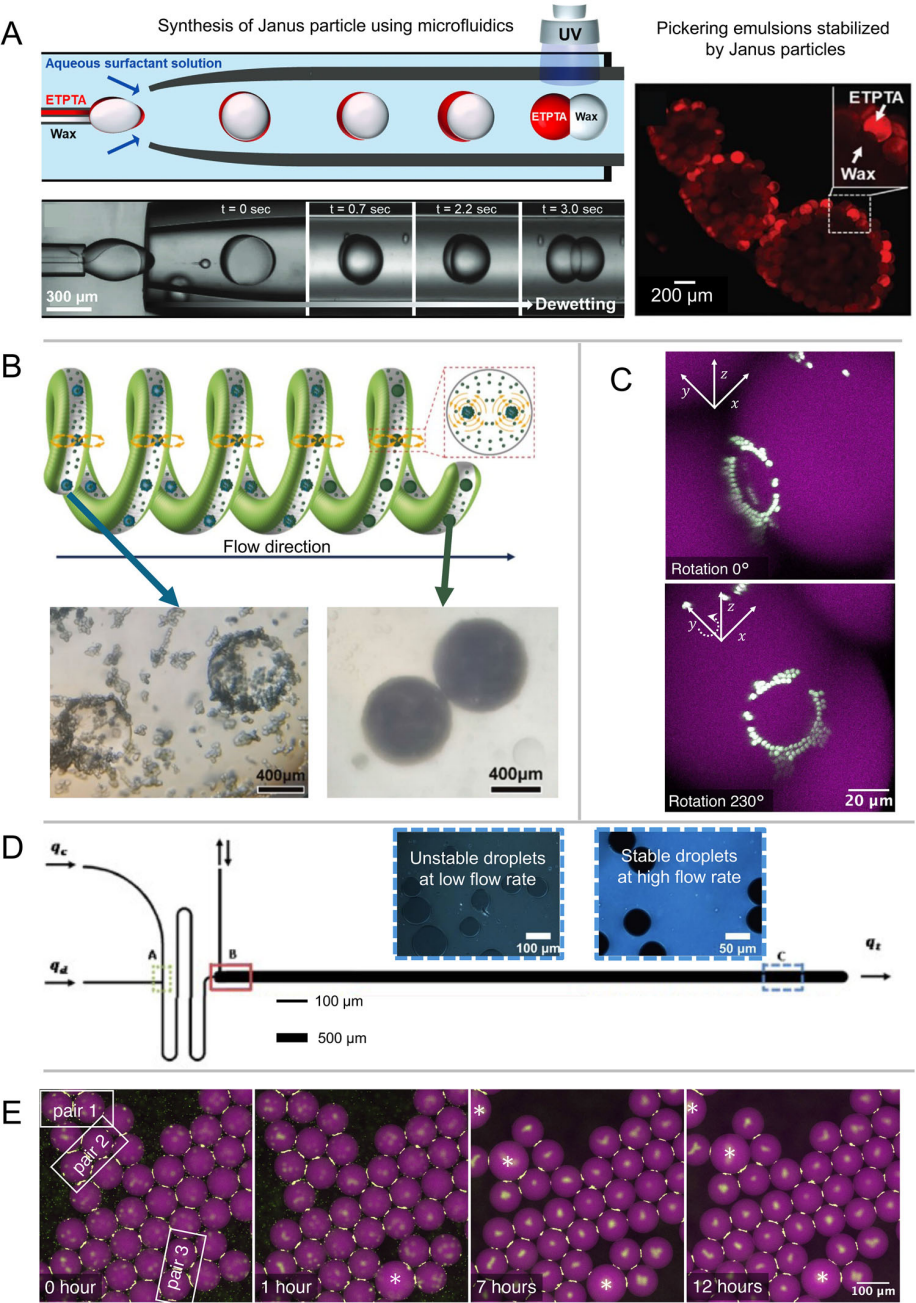
Applications of Microfluidics in Pickering Emulsions and Foams. A) Oil‐in‐water Pickering emulsions stabilized by Janus particles, synthesized using microfluidics. The Janus particles consist of a hydrophilic acrylate resin (ETPTA) and a hydrophobic paraffin wax, arranged in a dumbbell‐shaped structure. Adapted with permission.^[^ ^116^ ^]^ Copyright 2020, Wiley‐VCH. B) Microfluidics for encapsulation: Genipin‐crosslinked BSA‐stabilized water‐in‐water Pickering emulsions, enabling rapid release of ranitidine hydrochloride. Micrographs show nearly full droplet coverage after transfer through a helical coiled tube. Adapted with permission.^[^ ^83^ ^]^ Copyright 2024, Elesvier. C) Microfluidics provide an efficient platform for characterizing collected Pickering emulsions. A 3D reconstruction of multiphoton fluorescence confocal images reveals the formation of crown‐like particle bridges between adjacent droplets. Reproduced with permission.^[^ ^13^ ^]^ Copyright 2025, Wiley‐VCH. D) Microfluidics can be used to observe coalescence stability during emulsification. The microfluidic device, schematically illustrated here, enables direct observation of whether droplets remain stable or undergo coalescence depending on the continuous phase flow rate. Adapted with permission.^[^ ^80^ ^]^ Copyright 2018, Elesvier. E) Microfluidics can be used to assess the long‐term stability of collected Pickering emulsions. Micrographs show the coalescence behavior (coalesced droplets noted by white stars) of on‐chip generated Pickering emulsions over a 12‐hour period. Reproduced with permission.^[^ ^13^ ^]^ Copyright 2025, Wiley‐VCH.^[^ ^13^, ^80^, ^83^, ^116^ ^]^

Further research into the application of microfluidics for Janus particles and Pickering emulsions primarily focuses on the fabrication of Janus particles using microfluidic devices. This technique allows for precise control over particle composition. For example, Janus particles can be fabricated using two chemically distinct biopolymers and ionically cross‐linked hydrogels,^[^ ^115^ ^]^ or bio‐based and responsive microparticles,^[^ ^116^ ^]^ as shown in **Figure** **5**A. Microfluidic methods offer excellent control over particle anisotropy, enabling the formation of particles with dual compartments that exhibit distinct properties such as surface wettability.^[^ ^60^ ^]^ Janus particles produced via microfluidics have found applications in both medical and food industries.^[^ ^114^ ^]^ For instance, a recently developed microfluidic process enables the production of triple‐phase Janus particles that exhibit programmable release behavior and enhanced tumor inhibition compared to monophasic particles and free drug delivery.^[^ ^117^ ^]^ Conventional preparation methods for Janus particles from organic polymers and inorganic materials typically require harsh conditions, such as high temperatures and electric fields.^[^ ^118^ ^]^ Therefore, utilizing bio‐based materials is essential for expanding the application of Janus particles, especially in the food field.^[^ ^2^ ^]^

### 5.2 Encapsulation Strategies for Food and Medical Applications

Encapsulation technology plays a pivotal role in enhancing the stability, delivery, and functionality of active ingredients. Pickering emulsions integrated with microfluidics have attracted significant attention in food research, aiming to improve sensory properties, protect sensitive compounds (such as vitamins, antioxidants, and probiotics) from environmental stressors, and enable controlled release during digestion. Yang et al. developed a new microfluidic setup to prepare monodispersed and highly particle‐covered water‐in‐water Pickering emulsions from a pH‐responsive polyethylene glycol/phosphate aqueous two‐phase system. Surface coverage rate of 96% could be achieved by optimizing the length of a helical coiled adsorption tube and the properties of genipin cross‐linked bovine serum albumin (BSA) particles,^[^ ^83^ ^]^ as shown in Figure 5B. Similarly, the pharmaceutical and medical fields explore methods to encapsulate and deliver active ingredients effectively. To fine‐tune the drug release characteristics of microspheres, mesoporous silica nanoparticles can be incorporated into poly(lactide‐co‐glycolide) composite microspheres using a microfluidic preparation technique.^[^ ^71^ ^]^ This approach achieved a sustained release of encapsulated model drug over four months without any burst release, which demonstrates the system's potential for controlled drug delivery. Li et al. developed a microfluidic‐based Pickering emulsion polymerization method to synthesize uniform magnetic fluorescent microspheres for encapsulating quantum dots, which demonstrated good stability and potential for multiplex tumor marker detection.^[^ ^84^ ^]^

Given the advantages of microfluidic techniques in addressing issues related to droplet size, their application extends beyond single emulsions to more complex multiple emulsions for encapsulation. One notable food‐related example is alginate microgels with multiple oil cores, which were produced using a two‐step Pickering emulsion method in a droplet microfluidic device.^[^ ^119^ ^]^ The process began with forming a stable oil‐in‐water Pickering emulsion using cellulose nanocrystals in combination with calcium carbonate. Next, these stabilized droplets were encapsulated in alginate microgels using microfluidics, where the released calcium ions triggered internal gelation by cross‐linking with alginate. The system improved protection and sustained release of lipophilic compounds compared to conventional capsules. Recent work has demonstrated the preparation of food‐grade water‐in‐oil‐in‐water emulsions using microfluidic techniques to reduce sugar content in which the core/shell droplets achieved 100% encapsulation efficiency and two‐month stability due to the gelatinized starch acting as a Pickering stabilizer.^[^ ^70^ ^]^

### 5.3 Nanoparticle–Microbubble Hybrids for Medical Imaging and Therapy

There is an increasing demand for multifunctional medical agents with the capability to support multiple imaging and therapeutic techniques to improve diagnostic accuracy. Microbubbles are widely used in ultrasound imaging,^[^ ^120^ ^]^ while in optical, photoacoustic, and MRI applications, nanoparticles such as quantum dots and gold nanorods can be employed. However, developing nanoparticle‐incorporated microbubbles for in vivo ultrasound has its own challenges, including maintaining optimal size (1–7 µm) for vascular travel and ultrasound resonance, ensuring shell properties that balance ultrasound responsiveness, biocompatibility, and nanoparticle inclusion, as well as ensuring stability under physiological conditions. Microfluidic techniques offer solution to the problem of polydispersity and integrate all the complementary reagents. By combining solid nanoparticles with monodisperse perfluorobutane (PFB) microbubbles using pH‐controlled electrostatic interactions, Seo et al. developed a strategy for creating multifunctional medical imaging and therapy agents.^[^ ^121^ ^]^ The resulting nanoparticle–microbubble hybrids, produced via microfluidics, are detectable by low‐pressure ultrasound suitable for in vivo diagnostic and therapeutic applications. Additionally, Pickering stabilization can be employed to produce water‐in‐air‐in‐water structures, known as antibubbles, which have potential applications in developing more stable microbubbles for drug delivery, as well as ultrasonic imaging.^[^ ^122^ ^]^

### 5.4 On‐Chip Analysis of Pickering Emulsions

The advancement of microfluidic technology has recognized emulsified droplets as ideal microreactors based on their uniformity and efficient mass transfer and reaction rates. Each droplet serves as an independent microreactor with increased contact area between reactants and catalysts while ensuring uniform mixing. The enhanced interfacial stability and mass transfer efficiency between two phases make Pickering emulsions an effective platform for catalysis and biodegradation of biphasic reactions involving, e.g., sensitive biological materials like enzymes or bacteria, and supporting high‐performance analysis.^[^ ^123^ ^]^ While some analyses, such as evaluating long‐term in vitro drug release profiles, may require extended incubation outside the microfluidic device,^[^ ^71^ ^]^ other reaction and release behaviors can still be further investigated through on‐chip analysis for insights into localized processes.

Another advancement in droplet microfluidics is the on‐chip characterization of physical properties at small length scales, often in combination with other analytical tools, such as microscopy. Figure 5C presents a 3D reconstruction of multiphoton fluorescence confocal images of collected Pickering emulsions produced via lab‐on‐a‐chip setting, revealing that the stabilization of poorly covered droplets is due to the formation of crown‐like particle bridges between adjacent droplets.^[^ ^13^ ^]^ Microfluidic devices enable real‐time monitoring and precise analysis of rapid processes occurring during and after droplet formation, including droplet coalescence during emulsification (Figure 5D), particle adsorption (Figure 2B), assembly behavior of adsorbed particles in response to hydrodynamic forces, and long‐term coalescence stability (Figure 5E).^[^ ^13^ ^]^ In petroleum industry, microfluidics also serves as an effective research platform for Pickering emulsions. Through microfluidic experiments, Xu et al. demonstrated that injecting oil‐in‐water emulsions stabilized with both nanoparticles and surfactants improves enhanced oil recovery in harsh‐condition reservoirs by promoting droplet accumulation in high‐permeability regions.^[^ ^124^ ^]^ Microfluidic systems have been used to evaluate the stability of hydroxyapatite magnetic nanoparticle‐based Pickering emulsions and their responsiveness to magnetic fields, which is crucial for petroleum applications where emulsions with tunable stability are required.^[^ ^125^ ^]^

### 5.5 Upscaling of Microfluidic Platforms Toward Commercialization

Droplet microfluidics holds significant promise for industrial applications; however, scaling to such levels requires overcoming current production limitations. High‐throughput production systems such as STEP,^[^ ^126^ ^]^ and EDGE,^[^ ^101^, ^127^, ^128^ ^]^ which enable the simultaneous formation of multiple droplets, have improved food emulsion production rates. Nonetheless, the emulsifiers employed in these studies are primarily surfactants and proteins, necessitating further investigation into the applicability of Pickering particles.^[^ ^129^ ^]^

Additionally, to facilitate commercialization, materials used in device fabrication must be both robust and cost‐effective.^[^ ^130^ ^]^ In pharmaceutical research, microfluidic methods for generating drug‐releasing particles are often developed using materials such as PDMS, which are not easily transferable to more industrially suitable materials like thermoplastics. Another key consideration is that microfluidic devices are typically connected to auxiliary components such as pumping systems, microscopes, cameras, and sensors.^[^ ^131^ ^]^ For commercial adoption, these systems must be miniaturized or integrated with existing instrumentation. Forigua et al. have suggested that an integrated microfluidic platform could simplify device operation in non‐specialist settings, thereby enhancing the feasibility of large‐scale applications.^[^ ^130^ ^]^

## 6 Outlook

More than 10 000 and 3000 articles were published in 2024 with Pickering emulsion and Pickering foam as the respective keywords. Similarly, searches using “microfluidic” and “emulsion” or “microfluidic” and “foam” as keywords yield over 6000 and 3000 articles, respectively. In comparison, there is little overlap (fewer than 50 studies) combining Pickering emulsion/foam studies with microfluidics. While microfluidics has since long demonstrated its effectiveness in studying emulsions, its application to Pickering emulsions and foams has thus remains limited but is now rapidly growing. We believe the primary challenge lies in the initial step—efficiently generating stable particle‐stabilized droplets (or bubbles) and effectively loading the particles onto droplet or bubble interfaces. Here, we have systematically investigated the methodologies for preparing Pickering emulsions and foams using microfluidics and we believe this will help the community to make efficient use of the microfluidic platform.

Only with the successful preparation of Pickering emulsions and foams can microfluidic systems fully leverage their advantages, including precise controllability, integrability, in situ observation capabilities, and minimal energy and sample volume requirements. Upon successful preparation, these systems employ various droplet and bubble manipulation techniques and have shown promise in applications ranging from functional particle synthesis to emulsion and foam production and analysis. Future advancements in microfluidic technology could enable the execution of complex experiments while maintaining fabrication simplicity, facilitating broader adoption. Moreover, improvements in the sensitivity and specificity of integrated instruments and assays will allow direct analysis of emulsions and foams produced within microfluidic systems.

Concrete challenges remain in the widespread adoption of microfluidic technology for studying Pickering emulsions and foams, as we discussed above. Addressing these issues will drive further innovation and uncover new applications. Efforts to explore novel particle‐liquid combinations, developing robust microfluidic platforms, and investigating underlying physical principles will speed up large‐scale applications. Although several approaches have been proposed to improve particle adsorption, a standardized best practice has yet to be established. Furthermore, the integration of parallelization – which could significantly increase microfluidic system throughput – remains largely unexplored. Promising future directions include utilizing Pickering stabilization to generate water‐in‐air‐in‐water structures (antibubbles) for stable drug delivery microbubbles, applying droplet‐trapping techniques on microfluidic chips to monitor gastrointestinal digestion kinetics^[^ ^17^ ^]^ in food Pickering emulsion systems, and employing high‐throughput production systems such as EDGE and STEP to improve Pickering emulsion production rates. In the near future, research on particle‐stabilized emulsions and foams is expected to expand, incorporating innovative system designs and developments. Simultaneously, the field is likely to mature through the refinement and application of existing tools to address complex physical and chemical challenges. Microfluidics, as an ideal investigative platform, will facilitate a deeper understanding of underlying mechanisms and enable high‐throughput screening of formulation parameters, ultimately accelerating the development of novel materials with broad applicability across diverse fields.

## Conflict of Interest

The authors declare no conflict of interest.

## Author Contributions

X.S. and F.I.H. contributed equally to this work. All authors conceived this work and wrote the manuscript together. All authors have read and approved the final version for publication.
